# Characterization of the fatty acid profile in the ventral midbrain of mice exposed to dietary imbalance between omega-6 and omega-3 fatty acids during specific life stages

**DOI:** 10.1186/s13104-022-06175-0

**Published:** 2022-09-05

**Authors:** Nobuyuki Sakayori, Masanori Katakura, Susumu Setogawa, Makoto Sugita, Kazuto Kobayashi

**Affiliations:** 1 Department of Physiology and Oral Physiology, Graduate School of Biomedical and Health Sciences, Hiroshima University, Hiroshima, 734-8553 Japan; 2 Department of Molecular Genetics, Institute of Biomedical Sciences, Fukushima Medical University, Fukushima, 960-1295 Japan; 3 Japan Society for the Promotion of Science, Chiyoda-ku, Tokyo, 102-0083 Japan; 4 Laboratory of Nutritional Physiology, Department of Pharmaceutical Sciences, Faculty of Pharmacy and Pharmaceutical Sciences, Josai University, Saitama, 350-0295 Japan; 5 Department of Physiology, Osaka Metropolitan University Graduate School of Medicine, Osaka, 545-8585 Japan

**Keywords:** Omega-6, Omega-3, Fatty acid profile, Midbrain, Life stage

## Abstract

**Objective:**

Omega-6 (*n*-6) and omega-3 (*n*-3) polyunsaturated fatty acids (PUFAs) are essential nutrients. Dietary imbalance between these PUFAs, in particular high in *n*-6 PUFAs and low in *n*-3 PUFAs (*n*-6^high^/*n*-3^low^), is common in modern society. We have previously reported that C57BL/6 mouse male offspring derived from mothers exposed to an *n*-6^high^/*n*-3^low^ diet during the gestation had an augmented ventral midbrain dopamine system in adulthood; however, the fatty acid composition in this brain region has not yet been investigated. This follow-up study aims to characterize the fatty acid profile of the ventral midbrain of mice exposed to the *n*-6^high^/*n*-3^low^ diet during specific life stages.

**Results:**

*n*-6 PUFAs, especially linoleic acid, were increased in the ventral midbrain of offspring exposed to the *n*-6^high^/*n*-3^low^ diet during the gestation compared to those exposed to a well-balanced control diet throughout life. On the other hand, *n*-3 PUFAs, especially docosahexaenoic acid, were decreased in the ventral midbrain of offspring exposed to the *n*-6^high^/*n*-3^low^ diet during the gestation, lactation, or postweaning period compared to those exposed to the control diet throughout life. Thus, exposure to the *n*-6^high^/*n*-3^low^ diet in pregnancy increases linoleic acid and that in any life stage decreases docosahexaenoic acid in the offspring's ventral midbrain.

**Supplementary Information:**

The online version contains supplementary material available at 10.1186/s13104-022-06175-0.

## Introduction

Fatty acids are primary components of lipids and are mainly categorized into saturated fatty acids (SFAs), monounsaturated fatty acids (MUFAs), and omega-6 (*n*-6) and omega-3 (*n*-3) polyunsaturated fatty acids (PUFAs). In the brain, these fatty acids work as structural components of the cellular membrane and serve as signaling molecules [1]. Animals can biosynthesize SFAs and MUFAs, but most animals cannot synthesize *n*-6 or *n*-3 PUFAs. Thus, these PUFAs are essential nutrients that must be obtained from the diet.

Major dietary sources of *n*-6 and *n*-3 PUFAs are linoleic acid (LA) and α-linolenic acid (ALA), respectively [2, 3], but major components of *n*-6 and *n*-3 PUFAs in the brain are arachidonic acid (ARA) and docosahexaenoic acid (DHA), respectively [4]. Regarding *n*-6 PUFAs in the brain, a large amount of *n*-6 docosapentaenoic acid (*n*-6 DPA) is synthesized when dietary deficiency in *n*-3 PUFAs is prolonged [5]. Regarding the functional aspects of these PUFAs, LA, ARA, ALA, and DHA in the culture medium induce the proliferation and/or differentiation of embryonic neural stem cells [6–9], and maintaining appropriate and sufficient levels of these PUFAs in the cellular membrane is essential for proper brain development [10–12]. Even after birth, the supply of these PUFAs is also critical for postnatal brain growth [13, 14], and the composition of *n*-6 and *n*-3 PUFAs in the adult brain is thought to affect various behaviors [5]. The supply of ARA and DHA also appears to be involved in the prevention of the age-accelerated functional decline in the brain [4, 15]. This evidence clearly shows that *n*-6 and *n*-3 PUFAs are indispensable components of the brain in every life stage.

*n*-6 and *n*-3 PUFAs are generally competitive in various metabolic processes, and the *n*-6/*n*-3 ratios of PUFAs that compose our bodies and our diets warrant particular attention [5, 16]. Regarding recent nutritional trends leading to foods that are high in *n*-6 PUFAs and low in *n*-3 PUFAs [17, 18], life-long exposure to a diet high in *n*-6 and low in *n*-3 PUFAs (an *n*-6^high^/*n*-3^low^ diet) beginning from conception to adulthood increased the levels of ARA and *n*-6 DPA and decreased that of DHA in the offspring’s whole brain during the embryonic, early postnatal, and adult periods compared to the offspring’s whole brain exposed to a well-balanced control diet [11, 12]. In parallel with such changes in the brain fatty acid profile, life-long exposure to the *n*-6^high^/*n*-3^low^ diet resulted in increased sucrose intake in the offspring compared to the offspring exposed to the control diet [12]. As sucrose intake behavior is strongly mediated by the ventral midbrain dopamine system [19], these data indicate that increased and/or decreased levels of PUFAs in the adult brain might augment the dopamine system, which was also proposed by another study [20].

In addition to the well-accepted notion that *n*-6 and *n*-3 PUFAs that compose the adult brain control various behaviors [5], recent birth cohort studies have found that maternal dietary *n*-6 and *n*-3 PUFAs are relevant to offspring behaviors in childhood [21, 22]. In animal studies, we also found that increased levels of ARA and *n*-6 DPA and decreased levels of DHA in the embryonic and early postnatal whole brain of the offspring exposed to the *n*-6^high^/*n*-3^low^ diet [11] were completely recovered to control levels in the adult whole brain of the offspring exposed to diets adequate in ALA from the early postnatal period to adulthood [23]; nevertheless, maternal exposure to the *n*-6^high^/*n*-3^low^ diet limited to the gestation period similarly led to increased sucrose intake in the offspring [12]. Thus, it is likely that offspring in utero exposed to the *n*-6^high^/*n*-3^low^ diet show increased sucrose intake without significant changes in the fatty acid profile of the adult whole brain. However, the fatty acid composition in the ventral midbrain of these mice has not yet been investigated. To further expand the findings obtained in our previous studies, we investigated the level of each fatty acid in the ventral midbrain of offspring exposed to the *n*-6^high^/*n*-3^low^ diet during specific life stages.

## Main text

### Materials and methods

#### Animals

C57BL/6 J mice were obtained from Clea Japan Inc. (Tokyo, Japan) and housed at Fukushima Medical University under a standard 12-h light/12-h dark schedule. Food and water were available ad libitum. No material for environmental enrichment was used. Animal or cage location was not randomized. All mice (*n* = 55) were healthy throughout the experiment, and no mouse was excluded from the analyses (> 20% reduction of body weight was set as a humane endpoint).

### Diets

The control and *n*-6^high^/*n*-3^low^ diets [12] were manufactured by Clea Japan Inc. The control diet was based on the AIN-93G diet [24] and was formulated to contain the following components: 51.9486 g cornstarch, 1 g α-cornstarch, 10 g sucrose, 7 g soybean oil (Additional file 1: Table S1), 1.4 mg tert-butylhydroquinone, 20 g milk casein, 0.3 g L-cystine, 5 g cellulose powder, 3.5 g AIN-93G mineral mix, 1 g AIN-93 vitamin mix, and 0.25 g choline bitartrate per 100 g complete diet. The *n*-6^high^/*n*-3^low^ diet was produced by replacing soybean oil in the control diet with high-linoleic safflower oil (Additional file 1: Table S1), as previously reported [10–12, 23]. Consequently, the *n*-6^high^/*n*-3^low^ diet contained more LA (18:2*n*-6) and less ALA (18:3*n*-3) than the control diet (Table 1).

**Table 1:** Fatty acid composition of the diets

Fatty acid	Control diet	*n*-6^high^/*n*-3^low^ diet
14:0	0.1% ± 0.0%	0.2% ± 0.1%
16:0	11.7% ± 0.2%	8.1% ± 0.0%
18:0	3.5% ± 0.3%	2.6% ± 0.0%
20:0	0.4% ± 0.0%	0.4% ± 0.0%
22:0	0.4% ± 0.0%	0.3% ± 0.0%
24:0	0.1% ± 0.0%	0.1% ± 0.0%
18:1	24.1% ± 1.6%	15.5% ± 0.3%
20:1	0.2% ± 0.0%	0.2% ± 0.0%
18:2*n*-6	52.6% ± 1.0%	71.8% ± 0.4%
18:3*n*-3	6.7% ± 0.5%	0.6% ± 0.0%
Total SFAs	16.4% ± 0.2%	11.8% ± 0.1%
Total MUFAs	24.3% ± 1.6%	15.8% ± 0.4%
Total PUFAs	59.3% ± 1.5%	72.4% ± 0.3%
*n*-6/*n*-3	8.0 ± 0.5	115.2 ± 7.9

Fatty acid composition is shown as % of total fatty acids (*n* = 3 diets)

### Feeding paradigm

Female mice (*n* = 3/group) were fed either the diet from 11 weeks of age and bred with male mice at 13 weeks of age. The offspring were weaned at 3 weeks of age, and the male offspring (*n* = 8/group) were group-housed at 2–4 mice per cage and those in adulthood were used for the behavioral and histological experiments in the previous study [12] and for the fatty acid analysis in this follow-up study.

**Fig. 1:**
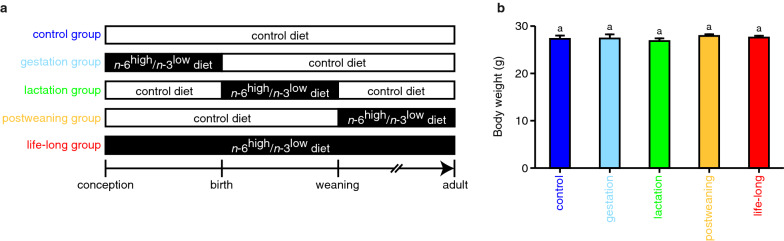
Experimental design to examine the effects of exposure to the *n*-6^high^/*n*-3^low^ diet during specific life stages on the fatty acid profile in the ventral midbrain in adulthood. **a** Feeding paradigm in the present study. **b** Body weight of the offspring. *n* = 8 mouse/group. Different letters show statistically significant differences (*P* < 0.05 by Tukey’s HSD test) between the groups

For life-long dietary exposure, we fed mother mice and their offspring with either the control diet or the *n*-6^high^/*n*-3^low^ diet throughout gestation, lactation, and postweaning periods, which were defined as the control group or the life-long group, respectively (Fig. 1a). We also fed them with the *n*-6^high^/*n*-3^low^ diet either during the gestation, lactation, or postweaning periods, and fed with the control diet during the remaining periods, which were defined as the gestation group, the lactation group, or the postweaning group (Fig. 1a).

### Fatty acid analysis of brain lipid fractions

The offspring that exposed to behavioral analyses [12] and maintained under free-feeding conditions were deeply anesthetized by intraperitoneal injection of sodium pentobarbital (> 75 mg/kg body weight), euthanized by cervical dislocation, and decapitated, and the brains were dissected from the surrounding tissues. To obtain the ventral midbrain, 1-mm-thick coronal sections were prepared using a brain slicer (BS-Z 2000C, Muromachi Kikai, Tokyo, Japan), and the ventral midbrain was manually dissected from the relevant section based on the mouse brain atlas [25]. The fatty acid composition of the ventral midbrains obtained from three offspring per group was evaluated using the gas chromatography as previously described [12, 26–28]. The gas chromatography was performed once. Three offspring data were averaged in different groups. Each peak area in the chromatogram was obtained through the data analysis software. The ratio of the peak area of each fatty acid to the peak area of the internal standard (tricosanoic acid) was calculated, and then the amount of each fatty acid in the analyte was calculated by multiplying the above ratio by the amount of the internal standard used. Recovery of the internal standard was 98.9% in average.

### Statistical analysis

To compare all the pairs of groups, Tukey’s honestly significant difference (HSD) test was used for each experiment by using SPSS Statistics (IBM, Armonk, NY). Differences with a *P* value < 0.05 were considered statistically significant. Normality of data was not evaluated. Sample size was determined based on our previous study [12]. The investigators were not blinded to the experiments. All summary data are presented as the mean ± SEM.

## Results

Mice in every group grew similarly, and we confirmed that their body weight in adulthood was similar (Fig. 1b).

**Fig. 2:**
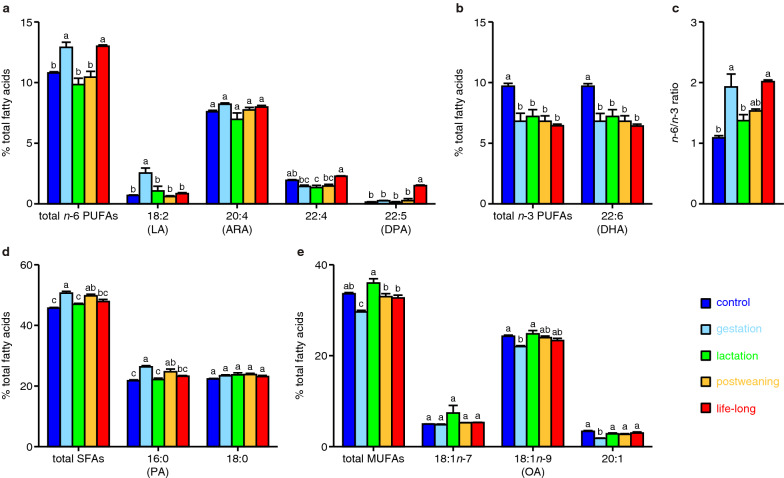
Fatty acid profile in the ventral midbrain of the offspring exposed to the *n*-6^high^/*n*-3^low^ diet during specific life stages. Levels of *n*-6 PUFAs (**a**), *n*-3 PUFAs (**b**), SFAs (**d**), and MUFAs (**e**) and the *n*-6/*n*-3 ratio (**c**) in the offspring’s ventral midbrain (*n* = 3 mouse/group). Fatty acids with more than 1% total fatty acids in either group are shown. Different letters show statistically significant differences (*P* < 0.05 by Tukey’s HSD test) between the groups

Next, the levels of *n*-6 and *n*-3 PUFAs in the ventral midbrain of these mice were evaluated (Additional file 1: Fig. S1 and Table S2). The levels of total *n*-6 PUFAs, especially LA (18:2) and *n*-6 DPA (22:5), were increased in the gestation and life-long groups, respectively, compared to the control group (Fig. 2a). The level of docosatetraenoic acid (22:4) was slightly decreased in the lactation group compared to the control group (Fig. 2a). Regarding *n*-3 PUFAs, the level of DHA (22:6) was decreased in every group (in the gestation, lactation, postweaning, and life-long groups) compared to the control group (Fig. 2b). We also found that the *n*-6/*n*-3 ratio in the ventral midbrain was increased in the gestation and life-long groups compared to the control group (Fig. 2c). These data show that the composition of *n*-6 or *n*-3 PUFAs in the adult ventral midbrain was differently or similarly affected, respectively, by exposure to an *n*-6^high^/*n*-3^low^ diet during specific life stages.

We also evaluated the levels of SFAs and MUFAs in the ventral midbrain (Additional file 1: Fig. S1 and Table S2). The level of total SFAs, especially palmitic acid (PA; 16:0), was increased in the gestation and postweaning groups compared to the control group (Fig. 2d). On the other hand, the total MUFA levels, especially oleic acid (OA; 18:1*n*-9) and to a lesser extent eicosenoic acid (20:1), were decreased in the gestation group compared to the control group (Fig. 2e). These data show that the composition of SFAs and MUFAs in the adult ventral midbrain was affected by gestational exposure to an *n*-6^high^/*n*-3^low^ diet and that of SFAs was also affected by postweaning exposure to an *n*-6^high^/*n*-3^low^ diet.

## Discussion

The level of DHA in the ventral midbrain may be differentially controlled compared to that in other brain regions. We have previously reported that exposure to the *n*-6^high^/*n*-3^low^ diet during the gestation period in mice decreased the level of DHA in the embryonic whole brain [11], which was totally recovered in the adult whole brain by postnatal exposure to diets adequate in ALA [23]. It has also been reported that exposure to an *n*-6^high^/*n*-3^low^ diet during the gestation period in rhesus monkeys decreased the DHA level in the frontal cortex at birth, but this reduction was completely recovered by postnatal exposure to a diet adequate in ALA for 2–3 years [29]. These reports suggest that reduced DHA in most brain regions can be recovered by postnatal dietary ALA in mice and monkeys. Thus, it is surprising that the DHA level in the ventral midbrain was decreased in every group in this study compared to the control group. Our data suggest that DHA in the ventral midbrain is vulnerable to reduction induced by dietary deficiency in *n*-3 PUFAs throughout life, and reduced DHA in the ventral midbrain that occurs in any life stage is not recovered by dietary ALA.

*n*-6 DPA is synthesized from adrenic acid, an *n*-6 PUFA, when dietary deficiency in *n*-3 PUFAs is prolonged and is thought to compensate for reduced DHA in the cellular membrane [5]. In line with this notion, we found that *n*-6 DPA and DHA in the ventral midbrain were increased and decreased, respectively, in the life-long group. However, in the gestation, lactation, and postweaning groups, *n*-6 DPA was not increased compared to that in the control group, despite reduced DHA. These results suggest that *n*-6 DPA does not play a role in compensating for reduced DHA in the ventral midbrain when exposure to an *n*-6^high^/*n*-3^low^ diet is limited to specific life stages.

Are any fatty acids in the ventral midbrain responsible for the increased sucrose intake in the offspring exposed to the *n*-6^high^/*n*-3^low^ diet? We have previously shown that offspring in the gestation and life-long groups, but not in the lactation or postweaning groups, showed increased sucrose intake compared to that of the control group [12]. However, in the present study, we did not find any fatty acids in the ventral midbrain that showed changes common in the gestation and life-long groups but not in the lactation or postweaning groups. As another possible mechanism by which exposure to the *n*-6^high^/*n*-3^low^ diet during the gestation period increased offspring sucrose intake in adulthood, we found that offspring in the gestation and life-long groups, but not in the lactation or postweaning groups, had an increased number of dopaminergic neurons in the ventral midbrain [12]. The production of dopaminergic neurons is only observed in the embryonic brain; thus, it is possible that increased dopaminergic neurogenesis in the embryonic midbrain caused by in utero exposure to an *n*-6^high^/*n*-3^low^ diet increases sucrose intake in adult offspring. The relationships between the levels of *n*-6 and *n*-3 PUFAs in the ventral midbrain and increased sucrose intake and between increased dopaminergic neurogenesis and increased sucrose intake warrant further investigations.

## Limitations

- We used the diets containing only ALA as an *n*-3 PUFA; however, *n*-3 PUFAs in human diets include not only ALA but also eicosapentaenoic acid and DHA [2, 3].
- The *n*-6/*n*-3 ratio in the *n*-6^high^/*n*-3^low^ diet is approximately 120/1, which is much higher than that in modern human diets, whose *n*-6/*n*-3 ratio is generally within the range of 4/1 to 20/1 [18]. Future study to investigate the effects of various dietary *n*-6/*n*-3 ratios on the brain fatty acid profile is warranted.
- We used male offspring in this study because female offspring exposed to the *n*-6^high^/*n*-3^low^ diet did not show increased sucrose intake compared to the female offspring exposed to the control diet [12]; however, we confirmed that there are sexual differences in the fatty acid profile of the whole brain between male and female mice [23]. Investigating the fatty acid profile of the ventral midbrain of female offspring exposed to the *n*-6^high^/*n*-3^low^ diet should be performed in future studies.

## Supplementary Information

**Additional file 1: Fig. S1.** Representative chromatograms obtained from the gas chromatography of the brain lipids. Chromatograms of the the control (**a**), gestation (**b**), lactation (**c**), postweaning (**d**), and life-long (**e**) groups. **Table S1.** Fatty acid composition of the dietary oils. Fatty acid composition is shown as % of total fatty acids (*n* = 3 dietary oils/group). **Table S2.** Retention time and peak area of each fatty acid in the chromatograms (*n* = 3 mouse/group).

## Data Availability

The datasets generated during this study are available from the corresponding author on reasonable request.

## Abbreviations

ALA: α-Linolenic acid
ARA: Arachidonic acid
DHA: Docosahexaenoic acid
DPA: Docosapentaenoic acid
HSD: Honestly significant difference
*n*-6^high^/*n*-3^low^: High in *n*-6 PUFAs and low in *n*-3 PUFAs
LA: Linoleic acid
MUFA: Monounsaturated fatty acid
OA: Oleic acid
PA: Palmitic acid
PUFA: Polyunsaturated fatty acid
SFA: Saturated fatty acid
