# Supplementary material for: Characterization of the fatty acid profile in the ventral midbrain of mice exposed to dietary imbalance between omega-6 and omega-3 fatty acids during specific life stages
Source: BMC Res Notes. 2022 Sep 5;15:285. doi: 10.1186/s13104-022-06175-0 (PMC9446585; doi:10.1186/s13104-022-06175-0)
Supplement: Supplementary file 1 — Additional file 1: Fig. S1. Representative chromatograms obtained from the gas chromatography of the brain lipids. Chromatograms of the the control (a), gestation (b), lactation (c), postweaning (d), and life-long (e) groups. Table S1. Fatty acid composition of the dietary oils. Fatty acid composition is shown as % of total fatty acids (n = 3 dietary oils/group). Table S2. Retention time and peak area of each fatty acid in the chromatograms (n = 3 mouse/group). [file 13104_2022_6175_MOESM1_ESM.pdf]

Fig. S1

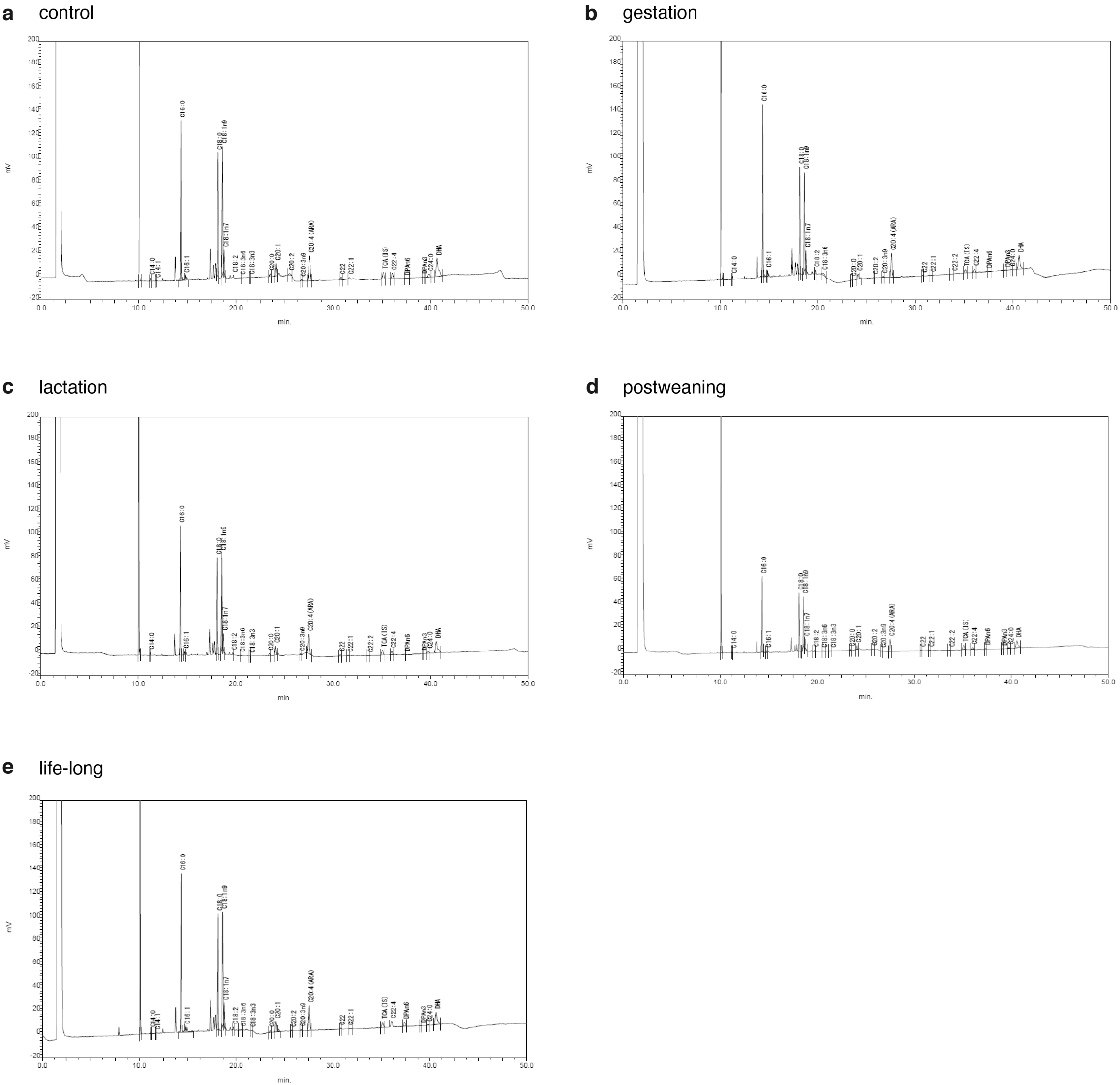

a control
b gestation
c lactation
d postweaning
e life-long
mV
min.
C16:0
C18:0
C18:1n9
C18:1n7
C20:4 (ARA)
TCA (IS)
DHA

Table S1

Fatty acid	soybean oil	safflower oil
14:0	0.1% ± 0.0%	0.1% ± 0.1%
16:0	10.5% ± 0.1%	6.9% ± 0.0%
18:0	3.4% ± 0.0%	2.7% ± 0.0%
20:0	0.3% ± 0.0%	0.3% ± 0.0%
22:0	0.4% ± 0.0%	0.3% ± 0.0%
24:0	0.0% ± 0.0%	0.0% ± 0.0%
18:1	26.6% ± 0.0%	16.2% ± 0.1%
20:1	0.2% ± 0.0%	0.2% ± 0.0%
18:2*n* -6	51.6% ± 0.3%	71.8% ± 0.2%
18:3*n* -3	6.3% ± 0.2%	0.8% ± 0.1%
total SFAs	14.8% ± 0.1%	10.3% ± 0.0%
total MUFAs	26.9% ± 0.0%	16.6% ± 0.1%
total PUFAs	58.3% ± 0.1%	73.1% ± 0.2%
n -6/*n* -3	8.3 ± 0.2	92.2 ± 5.9

Table S2

Fatty acid	retention time (min)	control	gestation	lactation	postweaning	life-long
16:0	12.36	455032.73 ± 51060.36	532331.87 ± 15709.31	480289.07 ± 45793.51	328828.00 ± 55660.81	434399.40 ± 45110.81
18:0	15.59	467699.47 ± 52885.39	472917.13 ± 8581.94	515458.33 ± 53833.51	312771.27 ± 37876.80	431044.60 ± 37541.03
18:1*n* -9	15.93	507880.53 ± 55156.57	445225.93 ± 10273.54	539266.13 ± 55889.34	316529.73 ± 44031.13	437239.13 ± 48737.02
18:1*n* -7	16.05	103989.80 ± 12195.08	97359.07 ± 4188.95	162035.27 ± 39624.85	69407.27 ± 9537.52	99339.80 ± 10246.59
18:2*n* -6	16.76	14406.80 ± 1631.60	51156.73 ± 7682.31	24518.13 ± 10761.18	7787.20 ± 702.10	15755.47 ± 2065.84
20:1*n* -9	20.16	70991.67 ± 10035.85	36819.33 ± 1426.70	60505.27 ± 3778.92	35755.13 ± 5113.34	56183.67 ± 7950.33
20:4*n* -6	22.79	158815.73 ± 17116.43	166115.60 ± 4498.11	149276.80 ± 5623.60	101459.47 ± 10983.71	149484.47 ± 16418.03
22:4*n* -6	29.80	40562.53 ± 4655.09	29039.67 ± 1859.37	28910.20 ± 3733.50	19279.67 ± 2609.83	42534.20 ± 4426.14
22:5*n* -6	30.83	2908.93 ± 683.56	5187.33 ± 373.75	2548.40 ± 1364.51	3262.73 ± 1789.45	28174.53 ± 3710.42
22:6*n* -3	33.70	204333.00 ± 28151.66	137448.53 ± 12272.56	154333.13 ± 3030.98	88748.47 ± 8920.15	120587.67 ± 15227.85
